# Announcment: Silicones for Pharmaceutical/Biomedical Applications

**DOI:** 10.1155/MBD.1995.295

**Published:** 1995


					TWO-DAY BIOMATERIALS SEMINAR IN BASEL
Silicones for
Pharmaceutical/Biomedical
Applications
14-15 NOVEMBER, 1995
HOTEL INTERNATIONAL, BASEL, SWITZERLAND
SPEAKERS
Alan L. Himstedt and Richard L. Gettings
Dow Corning Corporation, Midland, Michigan, U.S.A.
KEY TOPICS INCLUDE
Synthesis of fluids, gels, resins and gums
Formulation and manufacture ofelastomers
Fabrication and processing of elastomers
Emerging silicone/organic polymers
Biosafety of silicones
Bulk drugs
Fermentation processes
Parenteral packaging components
Tubing and fluid transport
Pharmaceutical excipients
Topical applications
Controlled drug delivery
Pressure sensitive adhesives
End-user applications/concerns
Emerging regulatory environment
Programme Division
TECHNOMIC Publishing AG, Missionsstrasse 44, CH-4055 Basel, Switzerland
Tel.: 061/381 52 26 Fax: 061/381 52 59
295

				

